# Optimal hyperglycemia thresholds in patients undergoing chemotherapy: a cross sectional study of oncologists’ practices

**DOI:** 10.1007/s00520-024-08756-0

**Published:** 2024-08-01

**Authors:** Teresa M. Salgado, Poorva B. Birari, Mona Alshahawey, Erin Hickey Zacholski, Emily Mackler, Tonya M. Buffington, Kerri T. Musselman, William J. Irvin, Jennifer M. Perkins, Trang N. Le, Dave L. Dixon, Karen B. Farris, Vanessa B. Sheppard, Resa M. Jones

**Affiliations:** 1 Department of Pharmacotherapy & Outcomes Science, School of Pharmacy and Massey Cancer Center, Virginia Commonwealth University, 410 N. 12Th Street PO Box 98053, Richmond, VA 23298 USA; 2 Department of Pharmacotherapy & Outcomes Science, School of Pharmacy, Virginia Commonwealth University, 410 N. 12Th Street PO Box 98053, Richmond, VA 23298 USA; 3 Department of Clinical Pharmacy, Faculty of Pharmacy, Ain Shams University, Organization of African Unity St, El-Qobba Bridge, El Weili, Cairo Governorate, 4393005 Egypt; 4 Michigan Oncology Quality Consortium (MOQC) and Michigan Institute for Care Management and Transformation (MICMT), 4251 Plymouth Road Arbor Lakes, Building 3, Floor 3, Ann Arbor, MI 48105 USA; 5 Bon Secours Mercy Health, 611 Watkins Centre Parkway, Suite 250, Midlothian, VA 23114 USA; 6 Emcara Health and PopHealthCare, 113 Seaboard Lane, Suite B200, Franklin, TN 37067 USA; 7 Bon Secours St Francis, 14051 St Francis Blvd Suite 2210, Midlothian, VA 23114 USA; 8 Division of Endocrinology, University of California San Francisco Medical Center, 400 Parnassus Ave., Suite A-550, San Francisco, CA 94143 USA; 9 Division of Endocrinology, Diabetes & Metabolism, Department of Internal Medicine, School of Medicine, Virginia Commonwealth University, 1101 E. Marshall St. Sanger Hall Suite 1-030, Richmond, VA 23298 USA; 10 Department of Clinical Pharmacy Translational Sciences, College of Pharmacy and Michigan Institute for Care Management and Transformation (MICMT), University of Michigan, 428 Church St, Ann Arbor, MI 48109 USA; 11 Department of Social and Behavioral Sciences, School of Public Health, and Massey Cancer Center, Virginia Commonwealth University, 830 East Main Street, Richmond, VA 23219 USA; 12 Department of Epidemiology and Biostatistics, College of Public Health, and Fox Chase Cancer Center, Temple University, 1301 Cecil B. Moore Avenue Ritter Annex, 9Th Floor, Suite 917, Philadelphia, PA 19122 USA

**Keywords:** Hyperglycemia, Oncologists [MeSH], Surveys and Questionnaires [MeSH], Practice Patterns, Physicians’ [MeSH]

## Abstract

**Purpose:**

Neither the United States nor the European oncology guidelines include details for appropriate management of hyperglycemia in cancer patients. The aim was to identify fasting and random blood glucose thresholds, and hemoglobin A1c (HbA1c) targets used by oncologists in clinical practice when managing hyperglycemia in patients with cancer undergoing chemotherapy.

**Methods:**

This national, cross sectional study utilized a questionnaire to collect oncologists’ perceptions about optimal blood glucose thresholds and HbA1c targets in patients with cancer undergoing chemotherapy. Descriptive statistics were calculated to summarize glucose thresholds, HbA1c targets, and sample characteristics. Responses to an open-ended question about oncologists’ approach to hyperglycemia management were analyzed via thematic analysis using an inductive approach.

**Results:**

Respondents (*n* = 229) were on average 52.1 years of age, 67.7% men, and 91.3% White. For patients without diabetes but experiencing hyperglycemia, oncologists targeted lower and upper fasting blood glucose levels between 75-121 mg/dL and 105-135 mg/dL, respectively. For patients with diabetes, the targets for lower and upper fasting blood glucose levels ranged between 100-130 mg/dL and 128-150 mg/dL, respectively. Fasting blood glucose (95.6%) and HbA1c (78.6%) were the most commonly used clinical indicators to consider chemotherapy dose reduction, delay, or discontinuation due to hyperglycemia in patients receiving chemotherapy with curative intent. Among those receiving palliative intent chemotherapy, the preferred clinical parameters were random blood glucose (90.0%), patient-reported blood glucose readings (70.7%), continuous glucose monitoring readings (65.1%), and patient-reported symptoms of hyperglycemia (65.1%). Three main themes emerged about oncologists’ approach to hyperglycemia management: 1) identification of high-risk patients; 2) need for early identification, screening, and diagnosis of hyperglycemia; and 3) multiple hyperglycemia management strategies.

**Conclusion:**

Oncologists reported a wide variation of target blood glucose ranges considered appropriate in patients undergoing chemotherapy. Lack of clear guidance for hyperglycemia management during chemotherapy in the United States may be contributing to a lack of consistency in clinical practice.

## Background

Hyperglycemia occurs in 20–60% of cancer patients as a result of the use of corticosteroids during chemotherapy treatment or due to specific anticancer agents, such as mammalian target of rapamycin (mTOR) inhibitors, phosphoinositide-3 kinase (PI3K) inhibitors, epidermal growth factor receptor (EGFR) inhibitors, immune checkpoint inhibitors, among others [1–9]. In addition to the risk of hyperglycemic episodes, patients undergoing chemotherapy are at an increased risk of developing new-onset diabetes and worsening control of their pre-existing diabetes [10–12]. Hyperglycemia can act as a prognostic factor of reduced overall survival and relapse-free survival [3, 4, 7, 13–18]. Compared to normoglycemia, hyperglycemia is associated with worse overall (*HR* = 2.05, 95% CI 1.67–2.51; P < 0.001) and disease-free (*HR* = 1.98, 95% CI 1.20–3.27; *P* = 0.007) survival [19]. This association is greater among individuals with pre-existing diabetes [19]. In a study examining hyperglycemic episodes in patients with advanced breast cancer with and without diabetes undergoing chemotherapy, diabetes with hyperglycemia was associated with decreased overall survival while having a diabetes diagnosis without hyperglycemia was not [14]. Furthermore, hyperglycemia can increase the risk of negative outcomes among cancer patients, including the risk of infections [20, 21], hospitalization [5], toxicity and morbidity [22, 23], and can induce tumor cell chemoresistance [24–26]. Hyperglycemia is also a risk factor for cancer recurrence [13] and may lead to chemotherapy dose reductions or early chemotherapy discontinuation [27].

Thresholds to define hyperglycemia in cancer patients vary significantly in the literature. Storey et al. [28] reviewed 30 studies that used a wide range of blood glucose levels to define hyperglycemia (e.g., 99 to < 126 mg/dL, greater than or equal to 126 mg/dL, greater than 200 mg/dL). Other studies used quartiles in accordance with the mean glucose levels beginning at 106 mg/dL, 106.7–117.2 mg/dL, 117.3–142.6 mg/dL, and ending at 142.6 mg/dL, while others described a classification of mild, moderate and severe, beginning at 121 mg/dL. Only seven of the 30 studies used the American Diabetes Association (ADA)’s criteria for diagnosing prediabetes and diabetes [28]. Diabetes is defined by the ADA as a fasting blood glucose equal or greater than 126 mg/dL, or a 2-h plasma glucose following an oral glucose tolerance test equal-to or greater than 200 mg/dL, or hemoglobin A1c (HbA1c) equal-to or greater than 6.5%, or a random plasma glucose equal-to or greater than 200 mg/dL in patients with symptoms of hyperglycemia [29].

Despite individual efforts to guide and educate oncologists and oncology nurses on how to manage diabetes and hyperglycemia in cancer patients [30, 31], it was not until May 2022 that a multidisciplinary team working on behalf of the United Kingdom Chemotherapy Board and the Joint British Diabetes Society for Inpatient Care created guidelines for the management of hyperglycemia in cancer patients with or without prior diabetes starting anticancer or glucocorticoid therapy [8, 32]. The document also provides guidance on identifying cancer patients who are at risk for hyperglycemic episodes and new onset diabetes mellitus [8, 32]. Neither the United States (US) nor the European oncology guidelines include details for the appropriate management of hyperglycemia in patients undergoing chemotherapy [33, 34]. In the US, the Common Terminology Criteria for Adverse Events (CTCAE) version 4.0 included specific blood glucose thresholds to classify hyperglycemia severity on a grade from 1–5 [35]. However, the currently used CTCAE version 5.0 presents a qualitative description of each severity level based on the medical and therapeutic interventions needed, without specifying any quantitative thresholds [35]. Furthermore, few package inserts for anticancer medications that are known to cause hyperglycemia include recommendations for dose-holding or reductions according to blood glucose level [36, 37]. Considering this relative lack of guidance, it is unclear what target blood glucose levels clinicians in cancer centers use in clinical practice when managing patients with cancer experiencing hyperglycemia.

Thus, the aim of this study was to identify target fasting and random blood glucose thresholds and HbA1c targets used by oncologists when managing hyperglycemia in patients with cancer undergoing chemotherapy and when making decisions about chemotherapy dose reduction, delay, or discontinuation.

## Methods

### Study design and participants

This study is a secondary analysis of data from a cross sectional, descriptive study aimed at assessing oncologists’ perceived responsibility, comfort, and knowledge managing hyperglycemia in cancer patients undergoing chemotherapy [38]. The study included a convenience sample of oncologists currently practicing in urban and suburban practices in the US (*n* = 229) recruited via Qualtrics. No exclusion criteria were applied. The sample size was based on a conservative estimation of an effect size of 0.075, 80% power, alpha = 0.05 and 10 predictors, which resulted in 226 respondents required for analysis. This study was classified as exempt by the Virginia Commonwealth University Institutional Review Board.

### Questionnaire and measures

The original questionnaire was developed by a multidisciplinary research team of oncology clinicians (i.e., one oncologist, two oncology pharmacists), two endocrinologists, two ambulatory care pharmacists, two behavioral scientists, a cancer epidemiologist, a health services researcher, and a clinical researcher. The questionnaire consisted of 46 items grouped into five sections: 1) responsibility for managing hyperglycemia during chemotherapy; 2) comfort in managing hyperglycemia during chemotherapy; 3) knowledge regarding hyperglycemia management during chemotherapy; 4) approach to hyperglycemia management; and 5) demographics and practice characteristics. The final version of the questionnaire and the results pertaining to the first three sections have been reported elsewhere [38]. The current manuscript focuses on section 4 – the oncologists’ approach to hyperglycemia management –, which included 16 questions. At the end, the survey included a box for open-ended comments about hyperglycemia management during chemotherapy treatment in patients with or without diabetes. Table 1 includes the measures used in this study. As explained in the introduction to the questionnaire, “hyperglycemia management during chemotherapy applies to all (single agent or combined) systemic anticancer treatment that patients receive, including infusion therapy, oral agents, and their accompanying supportive therapy in the outpatient setting.” A convenience sample of four oncology fellows (not part of the Qualtrics panel) pre-tested the questionnaire between May 30 – June 7, 2022, to establish face validity and to assess questionnaire understanding and flow [39]. Feedback regarding language clarity and response options was incorporated at the end of the process.

**Table 1:** Questionnaire items related to oncologists’ approach to hyperglycemia management

Questionnaire item	Coding
1. Do you look for guidelines to manage hyperglycemia outside of your specialty (e.g., American Diabetes Association, American Heart Association)?	Binary (Yes/No, if Yes specify)
2. Is there a protocol for hyperglycemia management at your institution/clinic?	3-level categorical (Yes/No/I don’t know)
3. What fasting blood glucose range (mg/dL) do you consider most appropriate for an oncology patient without diabetes experiencing chemotherapy-induced hyperglycemia?	Continuous
4. What is the fasting blood glucose range (mg/dL) do you consider most appropriate for an oncology patient with diabetes experiencing chemotherapy-induced hyperglycemia?	Continuous
5. What random blood glucose range (mg/dL) do you consider most appropriate for an oncology patient without diabetes experiencing chemotherapy-induced hyperglycemia?	Continuous
6. What random blood glucose range (mg/dL) do you consider most appropriate for an oncology patient with diabetes experiencing chemotherapy-induced hyperglycemia?	Continuous
7. What hemoglobin A1c target (%) do you consider most appropriate for an oncology patient without diabetes experiencing chemotherapy-induced hyperglycemia?	Continuous
8. What hemoglobin A1c target (%) do you consider most appropriate for an oncology patient with diabetes experiencing chemotherapy-induced hyperglycemia?	Continuous
9. What clinical indicator of hyperglycemia do you use to make decisions regarding dose reduction, delay or discontinuation of chemotherapy treatment with curative intent? Select all that apply	7-level categorical (a. fasting blood glucose, b. random blood glucose, c. hemoglobin A1c, d. patient-reported blood glucose reading, e. continuous glucose monitoring, f. patient symptoms of hyperglycemia e.g., polyuria, polyphagia, polydipsia, g. other, please specify)
*Display logic depending on which option(s) were selected in previous question*	
10. In general, at what fasting blood glucose level (mg/dL) do you consider dose reduction, delay or discontinuation of chemotherapy treatment with curative intent?	Continuous
11. In general, at what random blood glucose level (mg/dL) do you consider dose reduction, delay or discontinuation of chemotherapy treatment with curative intent?	Continuous
12. In general, at what hemoglobin A1c value (%) do you consider dose reduction, delay or discontinuation of chemotherapy treatment with curative intent?	Continuous
13. What clinical indicator of hyperglycemia do you use to make decisions regarding dose reduction, delay or discontinuation of chemotherapy treatment with palliative intent? Select all that apply.	7-level categorical (a. fasting blood glucose, b. random blood glucose, c. hemoglobin A1c, d. patient-reported blood glucose reading, e. continuous glucose monitoring, f. patient symptoms of hyperglycemia e.g., polyuria, polyphagia, polydipsia, g. other, please specify)
*Display logic depending on which option(s) were selected in previous question*	
14. In general, at what fasting blood glucose level (mg/dL) do you consider dose reduction, delay or discontinuation of chemotherapy treatment with palliative intent?	Continuous
15. In general, at what random blood glucose level (mg/dL) do you consider dose reduction, delay or discontinuation of chemotherapy treatment with palliative intent?	Continuous
16. In general, at what hemoglobin A1c value (%) do you consider dose reduction, delay or discontinuation of chemotherapy treatment with palliative intent?	Continuous
Please provide your comments about hyperglycemia management during chemotherapy treatment in patients with and/or without diabetes	Open-ended

Demographics and practice characteristics collected for sample description purposes included: age, gender, race, ethnicity, number of years in practice, sub-specialty, Oncology Board Certification, state where oncologists practiced, practice setting, practice size, practice location, and number of patients seen per week.

### Data collection

Data collection was performed between June 30, 2022 and August 30, 2022. Participants were recruited via email and questionnaires were administered over the telephone by Qualtrics’s sample partners. Informed consent was obtained prior to initiating the interviews. The questionnaire took no more than 15 min to complete, and responses were voluntary and confidential. Respondents received a $175 incentive.

### Data analyses

Descriptive statistics were calculated with means and standard errors (SEs) for continuous variables and frequencies and percentages for categorical variables. Because all responses were required, there were no missing data.

Violin plots were obtained to display upper and lower limits of fasting and random blood glucose levels and HbA1c targets indicated by oncologists in patients with and without a prior diabetes diagnosis. Violin plots combine a box plot and a kernel density plot. The width of the density curves is associated with the frequency of the data points in each area. The advantage of violin plots over box plots is that they allow visualization of the shape of the distribution, such as peaks, valleys, and bumps (i.e., clusters or data) in the density trace and they visibly depict clusters of data if the data distribution is multimodal [40].

Statistical analysis was performed using IBM SPSS software, version 26. Violin plots were created using the R Statistical Software (version 4.2.3; R Core Team 2023).

Responses to the open-ended question were analyzed via thematic analysis and using an inductive approach. Two independent researchers (MA and PBB) read all comments and generated initial codes in Microsoft Excel®. Subsequently, the authors grouped initial codes into broader themes and subthemes. Throughout the process, the two authors met to discuss the themes and subthemes and discrepancies were reconciled through discussion and consensus with a third investigator (TMS) using investigator triangulation [41]. Themes and subthemes were illustrated by presenting exemplar quotes.

## Results

The sample included 229 oncologists, mean age 52.1 years, an average of 22.6 years of experience, and with various subspecialites. Over two-thirds were men and 91.3% were White. Oncologists practiced predominantly in the Southern US (43.8%), in community oncology practices (40.2%), academic medical centers (28.8%), or both (31.0%), urban settings (97.4%), medium size practices (83.4%), and had a patient load of more than 76 patients per week (69.0%) (Table 2). Thirty-seven of 50 states were represented.

**Table 2:** Oncologist demographics and practice site characteristics (*n* = 229)

Characteristic	**Mean (SE)**
Age (years)	52.1 (0.64)
Years of experience as an oncologist	22.6 (0.62)
Characteristic	**n (%)**
Gender	
Man	155 (67.7)
Race/Ethnicity (more than one possible answer)^a^	
White/Caucasian	209 (91.3)
Hispanic or Latino	14 (6.1)
Black/African-American	5 (2.2)
Prefer not to disclose	1 (0.4)
Sub-specialty^b^	
Breast/Gynecological	61 (26.6)
Thoracic/Head and Neck	53 (23.1)
Gastrointestinal	34 (14.8)
Hematology	33 (14.4)
Genitourinary	24 (10.5)
Radiation/Surgical/Unspecified	13 (5.7)
Skin	11 (4.8)
United States region	
South	98 (42.8)
Midwest	50 (21.8)
West	44 (19.2)
Northeast	37 (16.2)
Type of practice site (more than one possible answer)	
Academic Medical Center	66 (28.8)
Community Oncology Practice	92 (40.2)
Both	71 (31.0)
Size of practice site	
Small (2–10 oncologists)	37 (16.2)
Medium (11–50 oncologists)	191 (83.4)
Large (> 50 oncologists)	1 (0.4)
Location of practice site	
Urban	223 (97.4)
Suburban	6 (2.6)
Patients seen per week	
< 50	12 (5.2)
51–75	59 (25.8)
76–100	73 (31.9)
> 100	85 (37.1)

^a^One respondent who identified as both White and Hispanic (question format was “select all that apply”) was recoded as Hispanic. One respondent who preferred not to disclose their race was coded as missing

^b^Breast/Gynecological includes breast, cervical, ovarian, uterine, vaginal; Thoracic/Head and Neck includes head and neck, lung, oral, oropharyngeal, thyroid; Gastrointestinal includes: colorectal, gall bladder, gastric, hepatic, pancreatic; Hematology includes hematology and leukemia; Genitourinary includes bladder, kidney, prostate; Radiation/Surgical/Unspecified includes general oncology, medical oncology, radiation, solid tumors, surgical oncology

### Approach to hyperglycemia management

The majority of oncologists (95.7%) reported looking for guidance to manage hyperglycemia outside of their specialty, and cited consulting the following information sources: American Association of Clinical Endocrinology, ADA, the United Kingdom National Health Service hyperglycemia guidelines, clinical trial publications, clinical research papers, endocrinology journals, and endocrine societies. All respondents indicated that there was a protocol for hyperglycemia management at their institution/clinic.

**Fig. 1:**
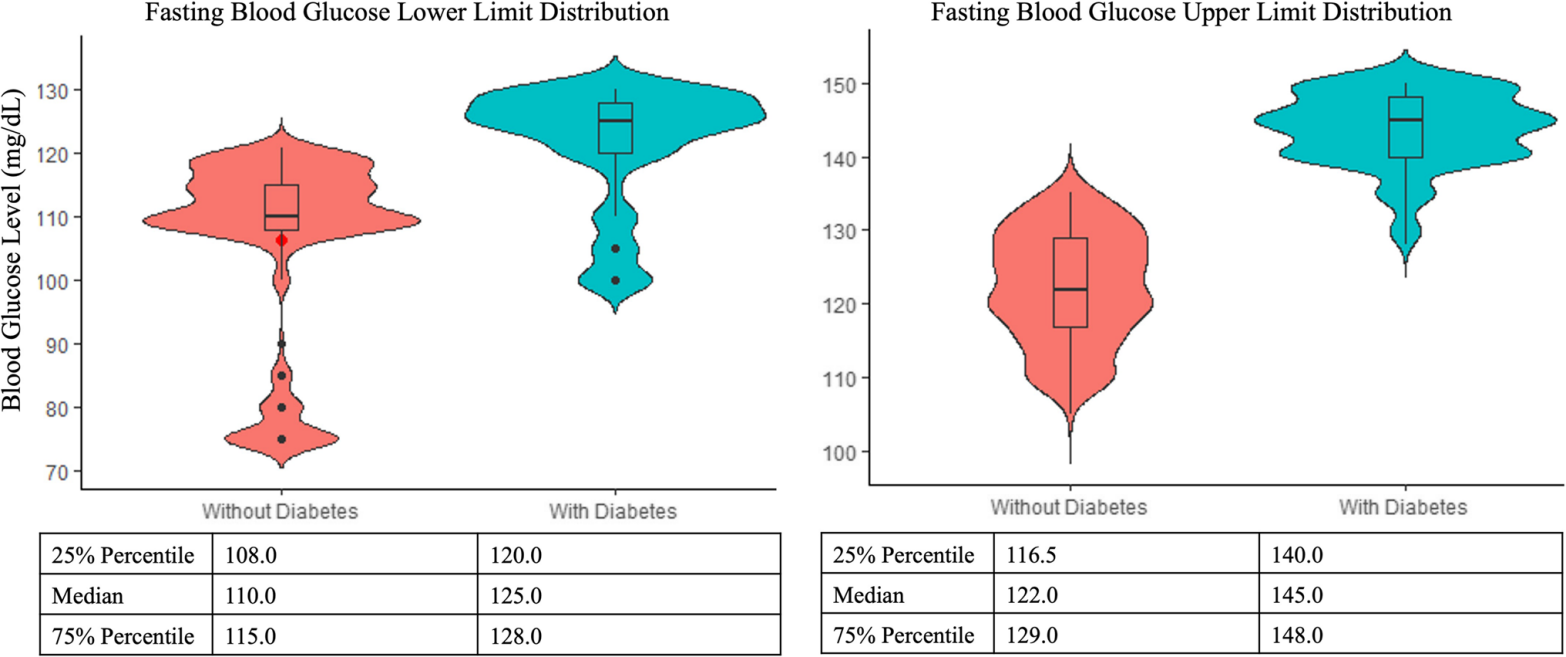
Violin plots displaying fasting blood glucose lower and upper limits distribution

**Fig. 2:**
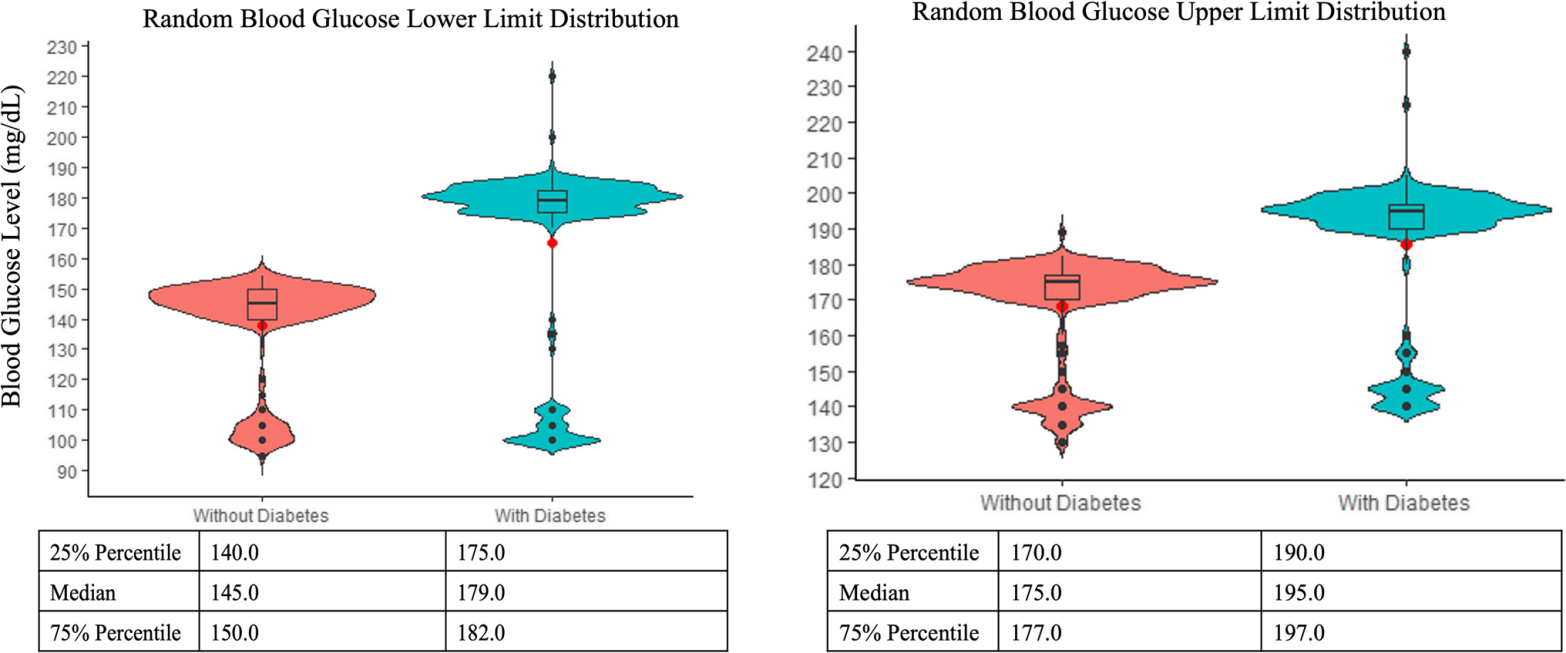
Violin plots displaying random blood glucose lower and upper limits distribution

**Fig. 3:**
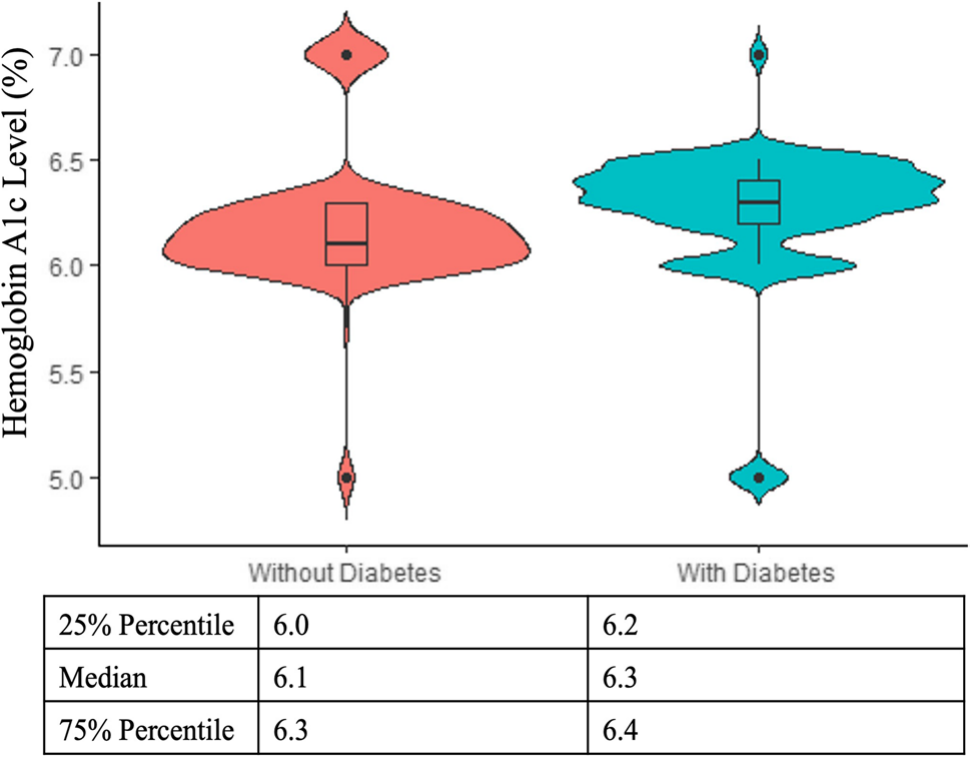
Violin plots displaying hemoglobin A1c distribution

The fasting and random blood glucose range and HbA1c target considered most appropriate in cancer patients with and without diabetes experiencing hyperglycemia varied greatly (Table 3). Violin plots show that oncologists considered that higher target fasting and random blood glucose levels were accepted in cancer patients with prior diabetes compared to those without (Figs. 1 and 2). Both figures depict a multimodal data distribution. Regarding fasting blood glucose levels, 18.8% oncologists indicated 130 mg/dL as the targeted lower limit and 22.3% considered 140 mg/dL the targeted upper limit in patients with prior diabetes. In patients without diabetes, 14.8% oncologists targeted 110 mg/dL as the lower limit and 16.2% targeted 120 mg/dL as the upper limit. Regarding random blood glucose levels, 17.9% oncologists indicated 180 mg/dL as the target lower limit, and 16.2% considered 200 mg/dL the target upper limit in patients with prior diabetes. In patients without diabetes, 18.8% oncologists targeted 150 mg/dL as the lower limit and 16.2% targeted 175 mg/dL as the upper limit. Despite a small difference in the mean and median HbA1c targets between cancer patients with and without diabetes, the data followed a multimodal distribution with 23.6% oncologists targeting HbA1c < 6.4% for cancer patients with diabetes and 27.1% oncologists targeting HbA1c < 6.0% for patients without diabetes (Fig. 3).

**Table 3:** Appropriate fasting and random blood glucose range (mg/dL) and hemoglobin A1c target (%) in oncology patients with and without prior diabetes experiencing chemotherapy-induced hyperglycemia

	Lower level		Upper level	
Without Diabetes	Mean (SE)	Min–Max	Mean (SE)	Min–Max
Fasting blood glucose	106.3 (0.96)	75–121	122.4 (0.49)	105–135
Random blood glucose	137.7 (1.17)	95–154	168.1 (0.98)	130–189
Hemoglobin A1c	–	–	6.2 (0.02)	5.0–7.0
With Diabetes	Mean (SE)	Min–Max	Mean (SE)	Min–Max
Fasting blood glucose	121.7 (0.62)	100–130	143.7 (0.33)	128–150
Random blood glucose	165.2 (2.01)	100–220	185.6 (1.37)	140–240
Hemoglobin A1c	–	–	6.3 (0.02)	5.0–7.0

Fasting blood glucose (95.6%) and HbA1c (78.6%) were the most commonly used clinical indicators to consider chemotherapy dose reduction, delay or discontinuation due to hyperglycemia in patients receiving chemotherapy with curative intent. Among those receiving palliative intent chemotherapy, the preferred clinical parameters were random blood glucose (90.0%), patient-reported blood glucose readings (70.7%), continuous glucose monitoring readings (65.1%), and patient symptoms of hyperglycemia (65.1%). The thresholds oncologists considered chemotherapy dose reduction, delay or discontinuation varied greatly (Table 4).

**Table 4:** Clinical indicators of hyperglycemia used by oncologists and level at which they consider dose reduction, delay or discontinuation of chemotherapy treatment with curative and palliative intents

	Oncologists using clinical indicators	Lower level^#^		Upper level^#^	
Chemotherapy treatment with curative intent	n (%)*	**Mean (SE)**	**Min–Max**	**Mean (SE)**	**Min–Max**
Fasting blood glucose	219 (95.6)	206.9 (6.55)	140–420	243.6 (7.19)	145–525
Random blood glucose	80 (34.9)	219.9 (4.71)	160–480	295.9 (4.53)	200–600
Hemoglobin A1c	180 (78.6)	–	–	6.5 (0.01)	6.0–7.0
Patient-reported blood glucose reading	29 (12.7)	–	–	–	–
Continuous glucose monitoring	87 (38.0)	–	–	–	–
Patient symptoms of hyperglycemia (e.g., polyuria, polyphagia, polydipsia)	97 (42.4)	–	–	–	–
Chemotherapy treatment with palliative intent	n (%)*	**Mean (SE)**	**Min–Max**	**Mean (SE)**	**Min–Max**
Fasting blood glucose	9 (3.9)	201.8 (37.49)	140–400	240.2 (51.50)	150–515
Random blood glucose	206 (90.0)	223.2 (5.90)	147–410	299.4 (6.66)	164–510
Hemoglobin A1c	6 (2.6)	–	–	6.5 (0.12)	6.2–7.0
Patient-reported blood glucose reading	162 (70.7)	–	–	–	–
Continuous glucose monitoring	149 (65.1)	–	–	–	–
Patient symptoms of hyperglycemia (e.g., polyuria, polyphagia, polydipsia)	149 (65.1)	–	–	–	–

^*^More than one possible answer, thus sum is greater than 100%

^#^Upper and lower levels collected for fasting and random blood glucose (mg/dL); target level collected for hemoglobin A1c (%) represented as upper level

### Free text comments about hyperglycemia management during chemotherapy

Three main themes emerged from the qualitative analysis regarding hyperglycemia management in patients undergoing chemotherapy: 1) identification of high-risk patients; 2) need for early identification, screening and diagnosis of hyperglycemia; and 3) multiple hyperglycemia management strategies (Table 5).

**Table 5:** Summary of themes and sub-themes identified from the open-ended comments and exemplar quotes

Theme	Sub-theme	Exemplar Quote
**Theme 1:** Identification of high-risk patients		*In my experience, patients receiving glucocorticoids or chemotherapy agents are more likely to develop Type 2 diabetes during their treatment. […] (Oncologist 1)* *Before beginning chemotherapy, patients should be examined for the following hyperglycemia risk factors: age, prediabetes, diabetes, and body mass index (BMI). According to American Diabetes Association (ADA) guidelines, diabetes is diagnosed based on fasting plasma glucose (FPG) and glycated hemoglobin (HbA1c) values. (Oncologist 100)* *High sugar levels are a major concern in obese patients undergoing chemotherapy. They have greater susceptibility due to the insulin resistance in their body. Cancer cells proliferate faster with negligible apoptosis in high glucose conditions. Therefore, obesity management is critical in patients to avoid hyperglycemia. (Oncologist 129)*
**Theme 2:** Need for early identification, screening and diagnosis of hyperglycemia		*Reduced onset and severity of hyperglycemia can be achieved by early identification of high-risk patients and attentive monitoring while they are receiving chemotherapy treatment. Many patients stop the anti-cancer treatment in between due to the discomfort caused by hyperglycemia and the ongoing treatment. […] (Oncologist 69)*
**Theme 3:** Multiple hyperglycemia management strategies	Multidisciplinary approach	*Every time there is a case of hyperglycemia during chemotherapy, I personally consider collaborating with an endocrinologist to manage the condition. Because of varied reasons like prior diabetes, comorbidities, age, *etc*. each patient's hyperglycemia management regime is different.* (Oncologist 219) *Management of hyperglycemia during chemotherapy involves a wide range approach that involves drugs to maintain the sugar levels, diet, exercise *etc*. Hence a diabetologist would be the best person to answer this question. It is beyond my scope of treatment.* (Oncologist 225) *I am primarily involved with giving chemotherapy at the outpatient infusion center. All patients with cancer get their HbA1c and a random plasma glucose checked prior to commencing anti-cancer treatment. Patients with a higher possibility of developing hyperglycemia are provided with capillary blood glucose meter and glucose testing strips. Patients with raised baseline HbA1c are referred to a primary care physician to manage the elevated blood sugar levels. (Oncologist 190)*
	Personalized hyperglycemia treatment	*There are no set methods for treating the illness. A wide range of challenges are involved with controlling hyperglycemia in a patient receiving continuous chemotherapy. It all boils down to employing a very adaptable, personalized approach based on what patients need and can handle. The patient should be instructed to closely monitor his or her blood glucose levels and maintain regular contact with the physician managing their diabetes. (Oncologist 12)* *I usually carry out the treatment in accordance with the situation of the patient. There are numerous strategies to treat hyperglycemia. For patients with mild hyperglycemia, strict carbohydrate control is very much effective. For managing patients with severe hyperglycemia my first choice is metformin and diet. (Oncologist 21)*
	Prescription of anti-diabetic medications	*[…] Patients who develop hyperglycemia for the first time as a result of chemotherapy are given metformin and advised to eat a healthy diet. (Oncologist 10)* *We start with hyperglycemia management in chemotherapy patients if the HbA1c levels are greater than 7%. Pharmacotherapy is usually what we go ahead with. Biguanides, which are insulin sensitizers are given to the patients and the dose is titrated based on blood glucose monitoring at intervals of 2–4 weeks. (Oncologist 8)* *The treatment of PI3Ki-induced hyperglycemia requires an integrative strategy that incorporates low-carbohydrate diets and drugs that lower blood sugar. Drugs that do not restrict the PI3K pathway are selected as primary and secondary agents for the treatment of hyperglycemia. Among these include metformin, thiazolidinediones, SGLT2 inhibitors, and glucosidase inhibitors. Due to its stimulatory effect on PI3K signaling, insulin should only be used as a last alternative for PI3Ki-related hyperglycemia. (Oncologist 52)*
	Regular monitoring	*I, along with my fellow diabetologist, manage hyperglycemia based on the grade of disease progression. For instance, if the patient has grade 1 hyperglycemia, we make sure that glucose level is monitored at least once a day and significant changes in the daily routine are recommended. Similarly, for grade 2 hyperglycemia, glucose level is monitored twice or three times a day and metformin therapy is initiated. (Oncologist 17)* *We always check patients' fasting blood glucose levels and hemoglobin A1c levels for hyperglycemia when giving chemotherapy and glucocorticoids. It is essential to screen and monitor every patient since hyperglycemia can affect even patients who are at minimal risk for it. When sugar levels are greater than normal, a patient should follow a regular regimen, balance their diet and be adherent to the treatment. (Oncologist 204)*
	Lifestyle modifications (diet, exercise, hydration, stress)	*Following a good diet is one of the best ways to manage hyperglycemia during chemotherapy. The diet should not contain high amount of carbohydrate be it simple or complex and even less protein. (Oncologist 19)* *In my opinion, managing hyperglycemia involves a lot of codependent factors. One of the most overlooked factor is mental health. Managing mental stress becomes very important in a situation like this, as glucagon level rises with increase in mental stress. Educating the chemotherapy patients on stress management, especially during instances of hyperglycemia is essential. (Oncologist 228)*
	Patient education	*All patients are well informed that hyperglycemia is prevalent during chemotherapy prior to the start of treatment. […] Patients are made aware of the symptoms and warning signs of hyperglycemia, including increased thirst, dry mouth, and frequent or heavy urination, and they are advised to contact the primary care physician if any of these symptoms appear. (Oncologist 30)* *We begin with the nurses educating patients on the condition. The nurses are trained to use simplified explanations especially while educating them on maintaining an optimal blood sugar levels. Understanding this helps the patients recognize the level of damage potential of poorly controlled blood glucose levels and encourages better adherence to the plan provided for managing their condition. (Oncologist 122)*

*PIKI3* phosphatidylinositol 3-kinase

*SGLT2* sodium-glucose cotransporter-2

#### Theme 1: Identification of high-risk patients

Oncologists highlighted the importance of identifying high-risk patients early on to implement appropriate management strategies and mitigate the risk associated with hyperglycemia. Patients receiving glucocorticoids and PI3K inhibitors were among those at a higher risk of developing hyperglycemia, along with patients with pre-existing diabetes, pre-diabetes, and obesity.

#### Theme 2: Need for early identification, screening, and diagnosis of hyperglycemia

Oncologists emphasized the importance of early hyperglycemia identification to prevent or minimize negative patient outcomes. They recommended that all patients receiving chemotherapy should be screened for hyperglycemia by means of fasting plasma glucose levels, HbA1c levels, or oral glucose tolerance tests before treatment initiation, and regularly during treatment. Oncologists voiced that screening should be individualized based on the patient's risk factors and that more frequent screening was warranted for high-risk patients (i.e., patients with pre-existing diabetes, pre-diabetes, obesity, receiving glucocorticoids, or PI3K inhibitors).

#### Theme 3: Multiple hyperglycemia management strategies

According to oncologists, managing chemotherapy-induced hyperglycemia was a determinant in how well patients responded to treatment. Oncologists listed several strategies to attain hyperglycemia management, including: a multi-disciplinary approach, treatment personalization, prescription of anti-hyperglycemic medications, regular monitoring, lifestyle modifications, and patient education.

### Multi-disciplinary approach

The need for a team-based strategy for managing chemotherapy-induced hyperglycemia was emphasized by oncologists who believed that it required the involvement of a range of healthcare professionals, including oncologists, endocrinologists, dietitians, and diabetes educators.

### Personalized hyperglycemia treatment

Oncologists highlighted the importance of personalizing hyperglycemia management to the unique needs and characteristics of each patient, including their medical history, concurrent medications, chemotherapy regimen, and glycemic status.

### Prescription of anti-hyperglycemic medications

The selection of anti-hyperglycemic medications should account for the patient's other comorbidities, consider potential drug-drug interactions with chemotherapy agents, and be adjusted based on the patient's response and glycemic control. Oncologists either implemented individualized patient treatment plans or followed recommended guidelines by the ADA or from their institution. Anti-hyperglycemic agents used included: insulin, biguanides, sodium-glucose cotransporter-2 (SGLT2) inhibitors, sulfonylureas, among others.

### Regular monitoring

Oncologists underlined that monitoring should take place on a frequent basis and be customized to the needs of each patient. Routine monitoring of blood glucose levels was viewed as critical for the early detection and treatment of chemotherapy-induced hyperglycemia by allowing for a prompt adjustment of insulin or other anti-hyperglycemic medications, while minimizing the risk of hypoglycemia or other side effects.

### Lifestyle modifications

Along with adjusting anti-hyperglycemic medications and routinely checking blood glucose levels, oncologists indicated that lifestyle changes were a crucial part of hyperglycemia management. They articulated that lifestyle changes could lower the risk of long-term complications, improve quality of life, and improve glucose management.

- Diet. Oncologists underlined the importance of a nutritious diet in enhancing glucose control, lowering insulin resistance, and enhancing general health. Oncologists advised patients to follow a healthy diet that was rich in lean protein, complex carbohydrates, and fiber, while avoiding processed, sugary, and high-fat foods.
- Physical activity. Regular physical activity, as part of a healthy lifestyle, was identified as an important strategy to improve glucose metabolism, enhance insulin sensitivity, and maintain overall glycemic control in cancer patients undergoing chemotherapy.
- Hydration. Oncologists recommended that patients maintained appropriate hydration and avoided caffeinated and sugary beverages. Additionally, they encouraged patients to monitor their fluid intake, particularly when undergoing chemotherapy, as some drugs might lead to dehydration.
- Stress management. Oncologists noted that stress reduction can promote general wellbeing by lowering insulin resistance, enhancing glucose control, and improving blood pressure. They suggested that patients engaged in stress-relieving routines such as yoga, meditation, or deep breathing. If needed, patients should also seek out social assistance and counseling.

### Patient education

Patient education was another aspect referred by oncologists in hyperglycemia management during chemotherapy. Patients were taught to identify symptoms of hyperglycemia (e.g., fatigue, excessive thirst, and frequent urination), how to manage symptoms, and how to manage medications.

## Discussion

In our previous work, oncologists perceived hyperglycemia management during chemotherapy to be outside of their scope of practice, considering it the responsibility of endocrinologists and primary care physicians (PCPs) [38]. This study highlights the wide variability of fasting and random blood glucose ranges and HbA1c targets that oncologists considered appropriate in patients undergoing chemotherapy. The range of accepted fasting and random blood glucose levels was even wider and higher when making decisions related to dose reduction, delay or discontinuation of chemotherapy treatment with both curative and palliative intents.

Among the 30 articles reviewed by Storey et al. describing the impact of hyperglycemia on health-related outcomes in patients with cancer, studies used a wide variety of glycemic thresholds to classify hyperglycemia and lacked consistency in the criteria used for diagnosing diabetes and pre-diabetes. While some studies used the ADA guidelines, a majority (*n* = 19) did not specify what criteria were used to define hyperglycemia [28]. In our study, 95.7% oncologists reported looking for guidelines to manage hyperglycemia outside of their specialty and indicated that their institution/clinic had a protocol to guide hyperglycemia management. However, it is unclear whether oncologists apply this information in clinical practice, as the variability in blood glucose thresholds and clinical parameters reported in our study is not consistent with current guidelines. Hyperglycemia management in clinical practice is complex and needs to account for patient comorbidities, as well as the impact of chemotherapy on appetite, kidney and liver functions.

In the US, CTCAE and medication package inserts can serve as the main oncology-specific clinical source for grading severity of hyperglycemia and making treatment decisions. Although the change in grading toxicity from blood glucose value in CTCAE version 4.0 to intervention needed in CTCAE version 5.0 may allow for better functional assessment of a patient’s toxicity from treatment, it also allows for subjectivity in blood glucose targets per institution and provider. This lack of objective guidance regarding blood glucose targets may partially explain the variability in practices that is patent in our results. Furthermore, few medication package inserts supply guidance for holding or discontinuing anticancer therapy based on blood glucose values. An exception to this is alpelisib, a PI3K inhibitor that carries a boxed warning for hyperglycemia caused by inhibition of intracellular response to insulin. The alpelisib package insert utilizes CTCAE version 4.0 criteria to guide initiation and intensification of anti-hyperglycemic treatment with metformin and insulin sensitizers, and recommends holding alpelisib at grade 3 toxicitiy (blood glucose > 250–500 mg/dL) [36]. Clinician recognition of these standard criteria were captured in free-text comments; therefore, clinicians may take a more standardized approach to management of alpelisib-induced hyperglycemia than otherwise captured in this study. Standardized blood glucose goals and management further allow for generation of real-world evidence. Alpelisib-induced hyperglycemia has been found to be more frequent in the real-world population than in clinical trials [42]. This finding is potentially due to strict exclusion criteria and hyperglycemia monitoring within clinical trials evaluating alpelisib that are not always replicated in the real-world population, and supports the pervasiveness of hyperglycemia in patients receiving chemotherapy and the utility of standard blood glucose targets in guiding management [43].

Regarding the parameters used to identify hyperglycemia, 12 studies in the review by Storey et al. used fasting blood glucose only, three used random blood glucose only, four used a combination of both, six used all available blood glucose values, five did not specify, and none used HbA1c [28]. A surprising finding from our study is the high proportion of oncologists (78.6%) who reported relying on HbA1c to base their decisions to reduce, delay, or discontinue chemotherapy due to hyperglycemia. This parameter is not regularly used in the cancer setting because anemia or the receipt of blood transfusions can alter its results and lead to inappropriate treatment [44]. Similarly, interpretation of HbA1c is also affected in patients with chronic kidney disease [45]. This represents an important opportunity for education of oncologists. Random and postprandial blood glucose, rather than fasting blood glucose, are the best measures to capture glucocorticoid-induced hyperglycemia [1, 46]. Time in range or glycemic variability obtained from continuous glucose monitoring provide better indication of glycemic control than HbA1c [47].

Our study has several implications for practice and research. Given discordance in guidelines reportedly used in clinical decision-making versus consistent blood glucose targets and HbA1c goals, future directions include exploring the need for resources that address oncology-specific hyperglycemia management. Development of oncology-specific guidelines or inclusion of patients with cancer as a special population within non-oncology guidelines could help to standardize management of patients with cancer and hyperglycemia. Further, it is unclear how the shift from a quantitative to a qualitative description of hyperglycemia severity in the CTCAE is perceived by oncologists and this aspect warrants future research. If the United Kingdom guidelines were to be adopted in the US, implementation in practice could still be an issue, as it takes an average of 17 years to translate evidence into practice [48]. Both the guidelines [8, 32] and the open-ended comments in our study allude to the need to have a multidisciplinary approach to hyperglycemia management, including endocrinologists, PCPs, nurses, pharmacists, and dietitians. As such, future research should also focus on understanding how the larger interdisciplinary oncology team (beyond oncologists) approaches hyperglycemia management and potential treatment discontinuation. Agreeing on thresholds for treatment discontinuation from an oncology standpoint would be helpful for diabetes practitioners (e.g., endocrinologists, PCPs) to promote more frequent visits with patients and more frequent dose adjstments.

In our previous study, oncologists viewed hyperglycemia management mostly as the endocrinologist’s responsibility, but the long wait times were cited as the greatest barrier to accessing these providers [38]. With the anticipated shortage of endocrinologists and PCPs in the US [49–51], it becomes critical to re-think the model of care that is provided to cancer patients. The use of telemedicine can be a means to increase access to endocrinologists [52, 53]. Alternatively, clinic-based pharmacists can function as care coordinators between the primary and secondary care settings [54]. Ambulatory care pharmacists are able to successfully manage diabetes under collaborative practice agreements and educate patients, which can be a great asset to the oncology team in need of more prompt support than an endocrinologist could provide [55–58]. Oral anticancer agents can cause or worsen diabetes, or interact with medications prescribed by PCPs [59], which is also an area that needs care coordination between oncology, endocrinology, and primary care. Furthermore, coordination with dietitians is critical to support cancer patients experiencing hyperglycemia.

This study has some limitations. First, Qualtrics recruitment did not result in any respondents practicing in rural areas. However, the sample was representative of the urban and suburban US oncology workforce with regard to age, gender, and race [60]. Second, oncologists working in the South constituted the majority of our sample. Importantly, the prevalence of obesity and diabetes is higher in the southern states compared to other US regions [61, 62]. In our previous work, however, the region of the US was not associated with any of the studied outcomes [38]. Third, and as a general limitation, we were unable to compare the characteristics of respondents and non-respondents, neither were we able to determine whether any oncologists were recruited from the same practice. Finally, potential mistakes during data entry cannot be ruled out because the questionnaires were interviewer administered; however, interviewers had standard training on interviewer-administered data collection. This limitation was also addressed by having interviewers share their screen with participants while they responded. Despite these limitations, our study highlights an important lack of uniformity in the approach to hyperglycemia management in patients undergoing chemotherapy among US oncologists.

## Conclusion

Oncologists reported a wide variability of fasting and random blood glucose ranges and HbA1c targets considered appropriate in patients undergoing chemotherapy. The target range of accepted fasting and random blood glucose levels was wider and tolerance for hyperglycemia was higher when making decisions related to dose reduction, delay or discontinuation of chemotherapy treatment with both curative and palliative intents. The lack of clear guidance for hyperglycemia management during chemotherapy in the US may be contributing to inconsistency in clinical practice.

## Data Availability

The datasets during and/or analyzed during the current study are available from the corresponding author on reasonable request.
